# Improvement of the Wear and Corrosion Resistance of CrSiN Films on ZE52 Magnesium Alloy Through the DC Magnetron Sputtering Process

**DOI:** 10.3390/ma18030536

**Published:** 2025-01-24

**Authors:** Hao-Yu Wu, Liang-Jyun Yang, Hou-Jen Chen, Shih-Hsien Chang, Hsin-Chih Lin

**Affiliations:** 1 Department of Materials Science and Engineering, National Taiwan University, Taipei 10617, Taiwan; f20513f20513f@yahoo.com.tw (H.-Y.W.); larry20010724@gmail.com (L.-J.Y.); f10527a06@ntu.edu.tw (H.-J.C.); 2 Department of Materials and Mineral Resources Engineering, National Taipei University of Technology, Taipei 10608, Taiwan; changsh@ntut.edu.tw

**Keywords:** RE-bearing magnesium–zinc alloy, magnetron sputtering, CrSiN thin film, wear, corrosion

## Abstract

The utilization of magnesium alloys as lightweight structural materials is becoming increasingly prevalent, particularly within the fields of electronics, automotive engineering, and defense. These alloys display high specific strength and excellent heat dissipation properties. The magnesium–zinc–rare earth alloy ZE52 displays superior formability and strength-ductility when compared to conventional magnesium alloys. A CrSiN film was deposited on the surface using a sputtering technique with the objective of enhancing wear and corrosion resistance for industrial applications. A CrSi buffer layer was deposited onto the ZE52 substrate prior to the deposition of the CrSiN film, with the objective of enhancing the adhesion between the two materials. The sputtering process for CrSiN films entailed the modulation of the substrate bias voltage. The CrSiN films exhibited a nanocomposite structure comprising CrN nanocrystallites embedded within an amorphous Si_3_N_4_, which resulted in enhanced hardness. Upon adjusting the bias voltage, improvements in mechanical properties were observed, with the film hardness and Young’s modulus increasing to 16.5 GPa and 187.4 GPa, respectively. Among the various CrSiN coatings under investigation, the ZE52 alloy that was coated with a CrSiN film deposited at a bias voltage of −50 V and a substrate temperature of 250 °C demonstrated the most favorable performance, exhibiting the lowest wear rate and superior corrosion resistance. In the tungsten carbide wear test with a loading of 4 N, the coating exhibited the lowest wear rate, at 2.2 × 10^−6^ mm^3^·m^−1^·N^−1^. Furthermore, the coating demonstrated remarkable corrosion resistance in a 3.5% NaCl solution, displaying a corrosion current density of 1.23 μA·cm^−2^ and a polarization resistance of 1271.4 Ω·cm^−2^.

## 1. Introduction

The demand for magnesium and its alloys is rising across a number of industries, including automotive, aerospace, electronics, and construction. This is due to the unique combination of properties that these materials possess. These materials exhibit a high strength-to-weight ratio, low density, and excellent thermal and electrical conductivity, which positions them as potential alternatives to traditional materials like steel and aluminum, particularly in moving components [1,2,3]. In the automotive sector, magnesium alloys are already utilized in components such as engine blocks, steering wheels, and transmission casings, driven by the growing need for lightweight structural materials to enhance fuel efficiency [1]. Despite the advantages of magnesium alloys, a significant limitation is their poor corrosion and wear resistance, which restricts their use in more demanding environments, particularly in components located near engine parts [4,5,6,7].

In order to address this challenge, the incorporation of rare earth (RE) elements into Mg-Zn alloys has emerged as an efficacious strategy for the improvement of corrosion resistance. The observed enhancement is largely attributed to the formation of metastable RE-rich phases along grain boundaries [8]. These rare earth (RE) elements interact with common impurities in magnesium alloys, such as iron (Fe), copper (Cu), and nickel (Ni), to form intermetallic compounds that mitigate micro-galvanic corrosion. The intermetallic phases possess low electrode potentials, reducing electrochemical activity with the magnesium matrix and significantly lowering corrosion rates. Consequently, the addition of RE elements plays a crucial role in enhancing the corrosion resistance and overall durability of magnesium alloys [9,10,11,12].

Rare earth (RE) elements also play a crucial role in improving the mechanical properties of magnesium alloys. These elements form intermetallic compounds with magnesium, often as micron-sized particles that remain stable even at elevated temperatures. This stability contributes to a reduction in the c/a ratio of the α-Mg phase [13], which mitigates the unfavorable basal texture characteristic of the hexagonal close-packed (HCP) crystal structure. Consequently, magnesium alloys demonstrate enhanced uniformity in deformation under mechanical loading, with the activation of additional slip systems that improve ductility [14]. The specific impact of rare earth (RE) elements on magnesium alloys is contingent upon the particular element employed. For example, a study conducted by Zhu et al. [15] examined the strengthening effects of incorporating yttrium (Y) and neodymium (Nd) into LA51 alloys. The findings indicated that yttrium facilitated more pronounced grain refinement. While both elements enhanced the alloy’s strength, yttrium improved elongation, whereas neodymium reduced it. Similarly, the incorporation of gadolinium (Gd) into magnesium–zinc alloys has been demonstrated to form thermally stable Mg_3_Gd phases, which promote further grain refinement [16]. In the study conducted by Tong et al. [17], different rare earth elements (Ce, Nd, and Y) were added to the AZ91 alloy for comparison to analyze their microstructure and mechanical properties. The results showed that alloys with rare earth element additions exhibited superior mechanical properties as a result of grain refinement. Specifically, the AZ91 alloy containing 1 wt% Nd demonstrated an increase in yield strength to 182 MPa and tensile strength to 264 MPa, while the AZ91 alloy containing 1 wt% Y achieved a yield strength of 184 MPa and tensile strength of 272 MPa. Both alloys not only increased the yield strength and tensile strength compared to the conventional AZ91 magnesium alloy but also exhibited significant improvements in hot temperature creep resistance. In addition, Zhou et al. [18] explored the effects of Nd and Y additions on the microstructure of the ZK60 alloy. Their findings revealed significant grain refinement, resulting in grain sizes of 90 μm, 60 μm, and 40 μm, respectively, which underscores the role of Nd and Y in enhancing the microstructural characteristics of magnesium alloys. Liu et al. [16] reported that the ZE53 magnesium alloy undergoes a T6 heat treatment process at 505 °C for 16 h, leading to the development of Zn_2_Zr_3_ precipitates. As a result, this transformation significantly improves the alloy’s mechanical properties, increasing the ultimate tensile strength, yield strength, and elongation to 280 MPa, 175 MPa, and 7.5%, respectively. The observed strength enhancement is attributed to the combined effects of second-phase strengthening and solid-solution strengthening mechanisms.

Notwithstanding the enhancements in mechanical and corrosion properties achieved through the addition of RE elements, these improvements frequently prove inadequate for demanding applications, particularly in the automotive industry. To meet these requirements, further advancements are required, particularly in the area of surface modification. The application of coatings via physical vapor deposition (PVD) has emerged as a promising solution. Research indicates that PVD coatings, including those of AlN, TiN, CrN, and TiAlN, significantly enhance the wear and corrosion resistance of magnesium alloys [19,20,21,22]. Compared to traditional coating methods, PVD provides superior durability and performance, making it a viable choice for demanding applications where enhanced surface properties are critical.

Chromium nitride (CrN) is a refractory transition metal nitride coating that has gained a reputation for its high hardness, chemical stability, and corrosion resistance, which have made it a popular choice for surface protection applications [23,24,25]. Although CrN is comparable to titanium nitride (TiN) in terms of hardness, as demonstrated in the study by Mansoor et al. [26], it exhibits superior electrochemical performance, with a lower corrosion current density and higher polarization resistance than TiN, indicating better corrosion resistance. Similar electrochemical results have also been reported in studies by Khan et al. [27] and Grips et al. [28]. Furthermore, Su et al. [29] revealed that although CrN has slightly lower hardness compared to TiN, its wear performance is significantly better, as demonstrated by smaller wear depths in both dry sliding and lubricated sliding tests. In addition, research by Broszeit et al. [30] demonstrated that CrN outperforms TiN in adhesion properties, further supporting its suitability for demanding industrial applications.

Nevertheless, in order to achieve even greater performance, chromium silicon nitride (CrSiN) has emerged as a promising alternative. In comparison to CrN, CrSiN exhibits enhanced resistance to wear, corrosion, and oxidation [31,32,33,34,35]. The incorporation of elements such as silicon (Si) or aluminum (Al) into CrN coatings has been demonstrated to markedly enhance their mechanical and tribological characteristics [35,36]. This improvement can be primarily attributed to the presence of an amorphous Si_3_N_4_ phase, which migrates to grain boundaries, inhibiting crystallite growth and thereby constructing a denser structure and fewer cracks and pinholes of the coating, increasing the coating’s hardness and better corrosion resistance [37,38]. Similarly, the properties of other binary nitride M-N films, such as TiN and ZrN, can also be enhanced by incorporating a third element (e.g., Si or Al) to form ternary nitride M-X-N films [39,40]. Diserens et al. [40] reported a nanocomposite structure consisting of nanocrystalline titanium nitride (TiN) embedded in an amorphous silicon nitride matrix (nc-TiN/a-Si_3_N_4_), which demonstrated improved hardness and abrasion resistance. The study conducted by Sandu et al. [37] indicated that in the ternary nitride M-X-N films, the segregation of X atoms on the MN crystallite surfaces limits their growth. This process results in the formation of an amorphous phase on the crystallite surfaces, further enhancing the mechanical properties of the coating. Therefore, the incorporation of silicon into CrN to form CrSiN coatings significantly enhances their performance by improving hardness, wear resistance, and corrosion protection. These advantages, driven by the formation of a denser structure and the inhibition of crystallite growth by the amorphous Si_3_N_4_ phase, establish CrSiN as a superior alternative to CrN for demanding surface protection applications. In order to optimize the corrosion and mechanical properties of CrSiN coatings, researchers have investigated a number of different Cr/Si chemical composition ratios [41], along with a variety of deposition parameters, including negative bias voltage (Vb) [33,34,35,36,37,41,42,43] and processing temperature (Ts) [34,35]. However, the majority of studies have concentrated on the application of CrSiN coatings to silicon substrates, with only a limited investigation of their behavior on magnesium alloys. This study addresses this gap by sputtering CrSiN films with a CrSi buffer layer onto ZE52 magnesium alloy substrates. The objective is to examine the effects of bias voltage (Vb) during the sputtering process, with the aim of enhancing the corrosion and wear resistance of ZE52 alloys through the optimization of CrSiN coatings.

## 2. Experimental Procedures and Methods

### 2.1. Substrate Preparation and Deposition Parameters

The magnesium–zinc–rare earth alloy ZE52, composed of 92.51 wt% Mg, 4.78 wt% Zn, 0.55 wt% Zr, 1.32 wt% Y, 0.43 wt% Gd, and 0.41 wt% Nd, was produced through direct-chill casting, followed by extrusion at 320 °C. The extruded alloys were initially cut into specimens measuring 200 × 100 × 10 mm^3^ and subsequently subjected to single-pass high-strain rate hot rolling, resulting in a reduction in the thickness to 2 mm. Subsequently, the rolled sheets were cut into specimens measuring 20 × 20 × 2 mm^3^. To prepare the surfaces, the specimens were subjected to a sequential grinding process utilizing #1000, #2500, and #4000 grit sandpaper. To achieve a scratch-free finish, polishing was conducted using aqueous suspensions containing 1.0 µm and 0.3 µm Al_2_O_3_ powder. Subsequent to polishing, the specimens underwent an ultrasonic cleaning process in acetone for 15 min and were then dried with high-purity nitrogen gas. To prevent oxidation, all specimens were stored in a vacuum environment maintained at 2 × 10^−3^ torr.

A direct-current (DC) magnetron sputtering system (LT-PVD400 model, Leitai Vacuum Co., Ltd., Taoyuan, Taiwan) was utilized to deposit CrSiN films onto ZE52 alloy substrates. The sputtering process employed a CrSi target with a diameter of 20 inches, comprising 90 at% chromium and 10 at% silicon, situated at an elevation of 80 mm above the substrate. In order to conduct a cross-sectional analysis, CrSiN films were also deposited onto silicon substrates. The deposition parameters for the CrSiN layers were as follows: a total deposition time of one hour, a sputtering power of 100 W, a working pressure of 0.009 torr, and a gas flow rate of 45/30 sccm for Ar/N_2_ (where sccm stands for standard cubic centimeters per minute). The deposition of the films was conducted at 250 °C with substrate bias voltages (Vb) spanning the range of −25 V to −75 V, as indicated by the labels −25 Vb, −50 Vb, and −75 Vb, respectively.

Nevertheless, the hardness of ZE52 alloys is approximately 60 HV, which is considerably lower than that of CrSiN (2500–3100 HV) [44]. This considerable disparity in hardness may result in inadequate adhesion between the substrate and the deposited layer [45]. Moreover, it is widely acknowledged that discrepancies in thermal expansion coefficients between the substrate and the sputtered film can result in film detachment due to temperature alteration. To address these limitations, a thin CrSi film was pre-deposited on the surface of the ZE52 alloy as an intermediary layer, effectively preventing film spalling caused by differences in hardness and thermal expansion coefficients. Furthermore, the incorporation of the CrSi layer mitigates internal stress at the interface with the substrate [46]. For the deposition of the CrSi layer, the total deposition time and argon flow rate were set to 15 min and 45 sccm of Ar gas, respectively, while all other parameters remained unaltered.

### 2.2. Characterization of CrSiN Coatings

The surface and cross-sectional morphologies of the CrSiN films were examined using a field emission scanning electron microscope (FEG-SEM, FEI Nova 450, Hillsboro, OR, USA). Phase identification was conducted via X-ray diffraction (XRD, Rigaku TTRAXIII 18 Kw, Tokyo, Japan) with Cu Kα radiation, employing a scan rate of 2°/min and a scan step of 0.02°. To reduce the impact of the substrate on the results, the incident X-ray angle was maintained at 0.8°. An atomic force microscope (AFM, Bruker Dimension ICON, Santa Barbara, CA, USA) was employed to analyze the surface at the nanoscale, providing high-resolution topographical images and quantifying surface roughness through the use of a sharp probe for scanning. The chemical states of the elements were determined via X-ray photoelectron spectroscopy (XPS, Thermo Fisher, Waltham, MA, USA). To assess the mechanical properties, a nanoindenter (Hysitron TI 980 TriboIndenter, Minneapolis, MN, USA) was utilized to quantify hardness, ensuring that the depth of indentation remained less than one-tenth of the film thickness to prevent substrate interference. To assess the adhesion between the substrate and the coatings, scratch tests were conducted using a scratch tester (Anton Paar Revetest RST 300, Graz, Austria) equipped with a diamond probe. The applied load ranged from 0 N to 20 N, with a scratch length of 5 mm. Subsequently, the surface morphology of the samples was examined through the use of optical microscopy (OM) and scanning electron microscopy (SEM). Wear resistance was evaluated through a pin-on-disk wear test apparatus (Freeform P.M. Co., Ltd., Kaohsiung, Taiwan) with a tungsten carbide ball (hardness: 90 HRA). The tests were conducted with loads of 2 N and 4 N, at a constant rotation speed of 0.2 m/s over 6000 cycles, with a 12 mm rotation diameter. The parameters of friction coefficient, wear volume loss, and wear rate were quantified. A field emission electron probe X-ray microanalyzer (FEG-EPMA, JEOL JXA-8530F PLUS, Tokyo, Japan) was employed to analyze the wear tracks, and quantitative composition analysis was performed using a wavelength dispersive spectrometer (WDS) to determine the chemical composition of both the coatings and the substrate. The depths of the wear tracks were measured with a precision instrument. Transmission electron microscopy (TEM) specimens were prepared using a focused ion beam (FIB) microscope (FEI Helios 600i, Hillsboro, OR, USA). Subsequently, a 200 kV field emission transmission electron microscope (FEG-TEM, FEI Tecnai G2 F20, Hillsboro, OR, USA) was employed to observe the microstructures of the CrSiN thin films. Electrochemical testing was conducted using a potentiostat (Autolab PGSTAT 204, Utrecht, The Netherlands) with a three-electrode electrochemical cell configuration in a 3.5 wt% NaCl aqueous solution at room temperature to assess the corrosion properties of the coatings. The experiments included potentiodynamic polarization (PDP) and electrochemical impedance spectroscopy (EIS). The specimen, with an active area of 2.01 cm^2^, served as the working electrode. A platinum electrode was employed as the counter electrode, while a saturated calomel electrode (SCE) served as the reference electrode. The PDP test was conducted with the open circuit potential (OCP) serving as the zero-reference point and a range of −0.4 V to 1.2 V at a scan rate of 1 mV/s. The polarization corrosion potential (E_corr_) and corrosion current density (I_corr_) were determined through the Tafel extrapolation method using Nova 2.1 software.

## 3. Results and Discussion

### 3.1. The Microstructure and Morphology of CrSiN Films Under Different Substrate Bias Voltage

**Figure 1:**
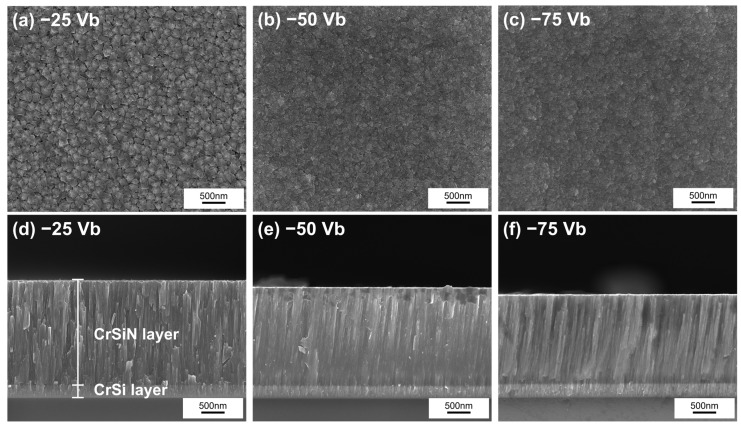
Surface morphologies of (**a**) −25 Vb, (**b**) −50 Vb, (**c**) −75 Vb CrSiN films and cross-sections of (**d**) −25 Vb, (**e**) −50 Vb, (**f**) −75 Vb CrSiN films.

Figure 1a–c illustrates the surface morphology of SEM images of CrSiN films deposited on ZE52 substrates with a CrSi buffer layer. Figure 1d–f displays the cross-sectional views of CrSiN films deposited on (100) silicon substrates. The thickness of each coating is provided in Table 1. The samples are designated as −25 Vb, −50 Vb, and −75 Vb, corresponding to substrate bias voltages (Vb) of −25 V, −50 V, and −75 V, respectively, and all deposited at 250 °C. The surface morphology images indicate that −25 V CrSiN films exhibit angular shapes with large, faceted structures, indicative of a rough surface. As the substrate bias voltage increases, the surfaces of −50 V and −75 V CrSiN films become progressively smoother and denser. This improvement can be attributed to increased ion energy, which enhances atomic mobility and results in denser films [42,43,44]. Cross-sectional SEM images further corroborate these findings. Increasing the substrate bias voltage significantly raises the energy of ionized plasma species, leading to more energetic impacts during film growth [47]. This elevated energy creates numerous defects in the growing film, which serve as heterogeneous nucleation sites [48]. Consequently, the number of nucleation points increases, resulting in higher grain density and reduced grain size. Simultaneously, the increased ion energy promotes surface densification and reduces surface porosity [33], producing finer columnar structures and narrower columnar grain widths [37]. This enhanced densification aligns with the improved surface smoothness observed in the surface morphology, ultimately reducing the CrSiN film thickness from 2.84 μm to 2.38 μm.

**Figure 2:**
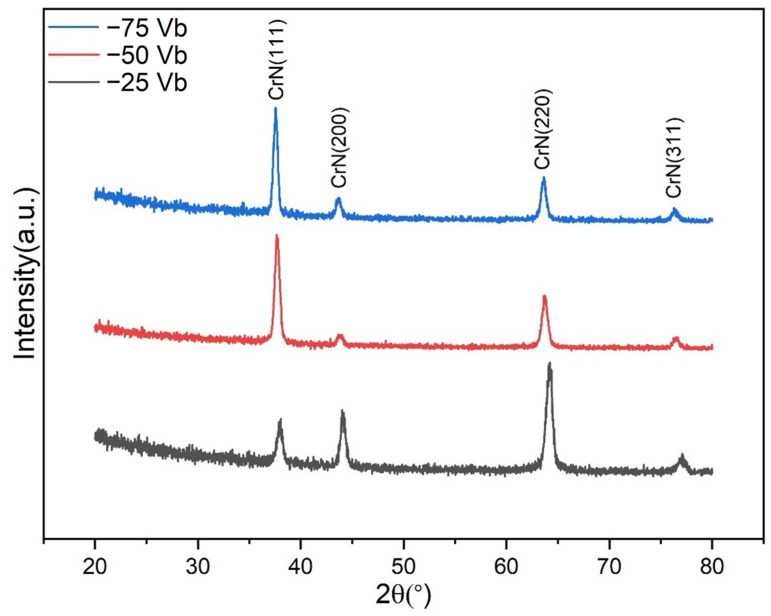
XRD patterns of CrSiN films deposited under different substrate bias voltages.

Figure 2 depicts the low-incidence-angle X-ray diffraction patterns of CrSiN films deposited under a variety of coating conditions. It is anticipated that the CrSiN films will comprise crystalline CrN and amorphous Si_3_N_4_. As illustrated in the results, the primary phases identified in all films were found to be cubic CrN, exhibiting diffraction peaks corresponding to the (111), (200), (220), and (311) planes. The lattice constants of the sputtered CrN peaks were calculated to be 0.4100, 0.4128, and 0.4136 nm for −25 V, −50 V, and −75 V CrSiN films, respectively. These values are all slightly lower than the standard lattice constant of 0.4140 nm for cubic CrN, as reported in [ICSD 00-011-0065]. This suggests that the tensile stress within the film increases with an increase in bias voltage. Conversely, no peaks corresponding to silicon-containing compounds (e.g., CrSi_2_, Si, or Si_3_N_4_) were identified, indicating that Si_3_N_4_ is likely present in an amorphous form [34]. Moreover, the films that were grown under conditions of weak ion irradiation at a substrate bias voltage of −25 V exhibited a predominant orientation of (220), with smaller fractions of (111), (200), and (311) planes. However, as the bias voltage increased to −50 V and −75 V, the films exhibited a transition to a dominant (111) texture. This transformation is attributed to the reconstruction of the crystal plane, which is driven by the elevated energy of the adatom, thereby facilitating the minimization of free energy. In accordance with the standard film growth model, the preferential growth of grains on low-energy surfaces is driven by the objective of minimizing surface energy. At a substrate bias voltage of −25 V, ions impart momentum to the coating surface, thereby promoting diffusion and adatom rearrangement, which in turn leads to significant strain accumulation. To reduce this strain, adatom alignment occurs on less stressed planes, such as the (220) plane in face-centered cubic (FCC) structures, due to its lower packing density, which generates less strain compared to denser planes [36]. However, at higher bias voltages of −50 V and −75 V, the preferred orientation shifts abruptly from the (220) plane to the (111) plane. This transition is attributed to the higher energy and diffusion rate of adatoms, which facilitate their rearrangement onto planes that minimize the overall surface energy, with the (111) plane being favored due to its lowest surface energy in FCC structures [49].

**Figure 3:**
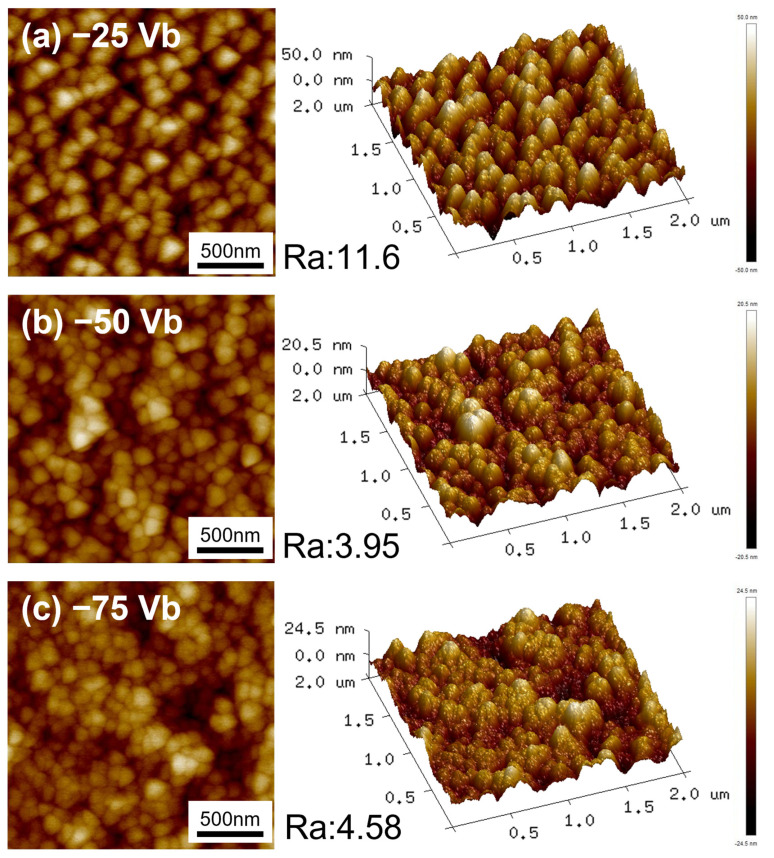
AFM surface morphologies and three-dimensional topographic images of (**a**) −25 Vb, (**b**) −50 Vb, (**c**) −75 Vb CrSiN films.

Figure 3 depicts the AFM surface morphologies and three-dimensional topographic images of CrSiN films under varying substrate bias voltages, accompanied by the corresponding surface roughness (Ra) values. At the lowest applied bias voltage of −25 V, the three-dimensional topographic images demonstrate the presence of relatively large grains and a surface that is characterized by a high degree of roughness. As the bias voltage increases to −50 V, the Ra value decreases, which can be attributed to enhanced atomic mobility and ion bombardment caused by the higher ion energy, leading to a reduction in surface particle size [50]. Furthermore, the reduction in roughness is associated with a reduction in surface defects and an improvement in film densification [51], which is consistent with the SEM surface morphology observations in Figure 1. However, when the bias voltage is increased to −75 V, the Ra value exhibits a slight increase compared to −50 V CrSiN films. This is attributed to more intense ion bombardment at higher bias voltages, which induces surface defects and results in greater surface undulations [43].

### 3.2. The Chemical Compositions and Bonding State of CrSiN Films Under Different Substrate Bias Voltages

**Figure 4:**
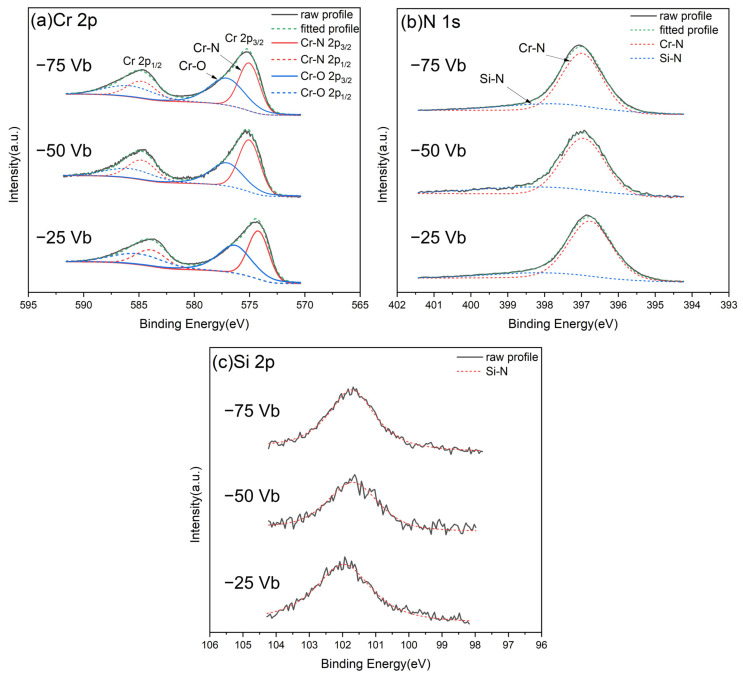
The curve-fitted XPS spectrum of CrSiN films under varying substrate bias voltages: (**a**) Cr 2p, (**b**) N 1s, and (**c**) Si 2p.

The chemical compositions of CrSiN films deposited at varying substrate bias voltages were analyzed using WDS, and the results are presented in Table 2. The data indicate that the Si/(Cr + Si) ratio consistently ranges from 0.10 to 0.11, while the nitrogen content remains between 45.56 and 46.6 at%. These findings indicate that varying the substrate bias voltage has a negligible effect on the elemental composition of the films. The presence of oxygen in the films is attributed to residual oxygen involved in the sputtering process or remaining gases within the spectrometer chamber. To gain further insight into the chemical states of CrSiN films under varying substrate bias voltages, high-resolution XPS analysis was conducted to investigate the near-surface characteristics. A Gaussian model was employed to fit all XPS spectra, thereby facilitating the acquisition of detailed chemical bonding information. The spectra were calibrated using the C 1s peak at 284.4 eV, as previously reported [52]. As illustrated in Figure 4a, the Cr 2p spectra of all films exhibit two discernible components, which are attributed to the Cr-N and Cr-O bonds, respectively. The lower binding energy peaks at 574.73 eV, 575.13 eV, and 575.08 eV, observed for substrate bias voltages of −25 V, −50 V, and −75 V, respectively, are associated with Cr-N bonds. These binding energy values are consistent with those reported in previous studies (574.5~575.3 eV) [53,54,55]. The higher energy value at 576.73~576.93 eV corresponds to Cr-O bonds, indicating that some Cr atoms are bonded to oxygen on the film surface [53]. As illustrated in Figure 4b, the N 1s spectra exhibit a broad peak at 396.83~397.08 eV. This peak can be deconvoluted into two components: a dominant peak corresponding to Cr-N bonds (396.73~397.03 eV) and a weaker peak attributable to Si_3_N_4_ bonds (397.88~398.08 eV) [55]. These findings substantiate the formation of both Cr-N and Si-N bonds in the films. Figure 4c depicts the Si 2p spectra for films deposited at varying substrate bias voltages. The fitted curves indicate the presence of exclusively Si-N bonds, with no discernible evidence of other potential bonds such as Si-Si or Si-O [54,56,57]. This finding strongly supports the conclusion that silicon exists predominantly in the form of a Si_3_N_4_ compound. Although XRD analysis is inherently limited in its ability to identify amorphous phases, the high-resolution XPS spectra provide definitive confirmation of the Si_3_N_4_ bonds, thereby substantiating its incorporation within the film matrix.

### 3.3. TEM Observation of CrSiN Films Under Different Substrate Bias Voltages

**Figure 5:**
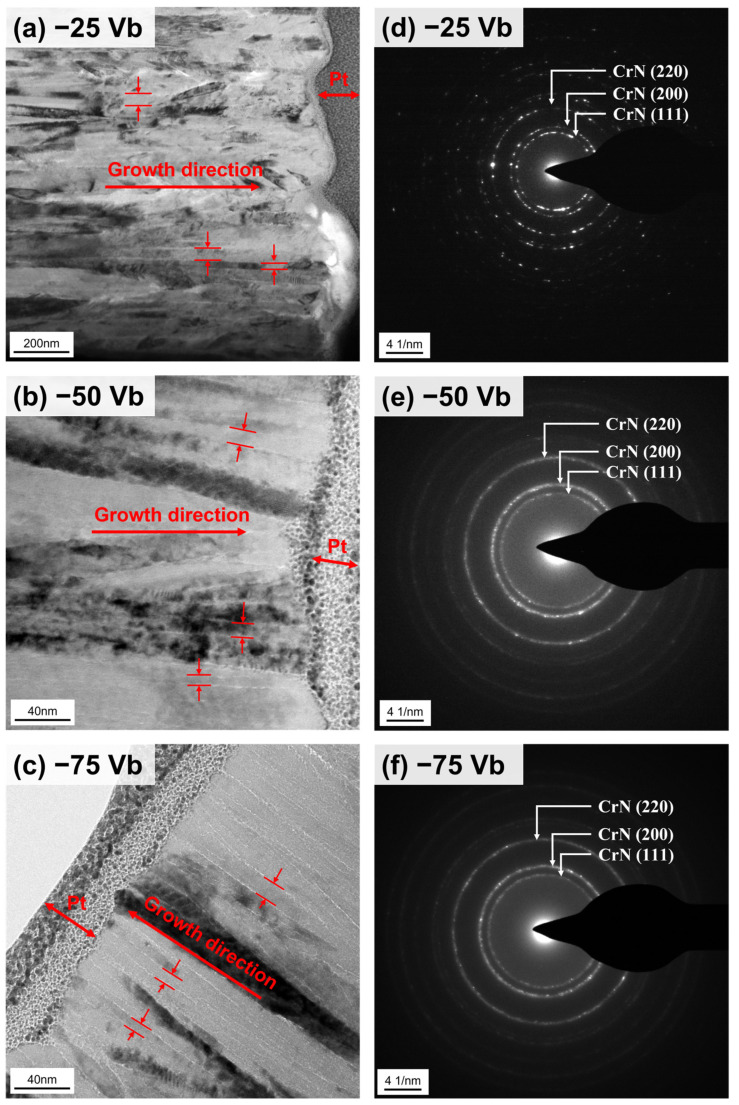
Cross-sectional bright field TEM images and the corresponding SAD patterns of CrSiN films deposited of (**a**) −25 V, (**b**) −50 V, and (**c**) −75 V, respectively. Diffraction rings for {111}, {002} and {022} are indexed in the SAD patterns of (**d**) −25 V, (**e**) −50 V, and (**f**) −75 V, respectively.

Figure 5a–c depicts cross-sectional TEM images of sputtered CrSiN films, where a protective Pt layer was deposited on top of the films. These images reveal dense columnar grain structures within the CrSiN films. As depicted in Figure 5, the columnar grains grow vertically relative to the substrate surface, as marked by the red arrow. Based on the TEM images, the average columnar grain widths for films deposited at substrate bias voltages of −25 V, −50 V, and −75 V are measured at 29 ± 4 nm, 9 ± 1 nm, and 8 ± 2 nm, respectively. Furthermore, the surface of the film deposited at −25 V displays a notable degree of roughness. The corresponding selected area diffraction (SAD) patterns for the three coatings are also presented in Figure 5d–f. In the SAD patterns of the CrSiN coating deposited at −25 V, discrete diffraction reflections are observed. In contrast, near-continuous segments of diffraction rings are present in the SAD patterns of the coatings deposited at −50 V and −75 V. This behavior suggests that the smaller columnar grain sizes and more crystal orientations in the −50 V and −75 V CrSiN coatings.

Furthermore, the transmission electron beam carries information from the three-dimensional structure. Upon passing through the CrSiN film, the amorphous Si_3_N_4_ invariably includes signals from crystalline CrN. This overlap presents a challenge in obtaining HRTEM images or FFT patterns of amorphous Si_3_N_4_. However, XPS analysis confirms the presence of amorphous Si_3_N_4_ within the matrix, which significantly contributes to the reduction in columnar grain size.

### 3.4. Mechanical and Wear Properties of CrSiN Films Under Different Substrate Bias Voltages

#### 3.4.1. Nano-Indentation Test of CrSiN Films

**Figure 6:**
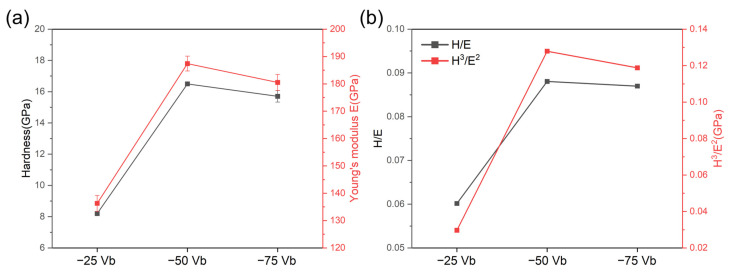
(**a**) Hardness (H) and elastic modulus (E), and (**b**) H/E and H^3^/E^2^ values of CrSiN films deposited under different substrate bias voltages measured by Nano-indenter.

Figure 6 and Table 3 summarize the nano-indentation test results for CrSiN films deposited at various substrate bias voltages. The data encompass measurements of hardness, elastic modulus, as well as the calculated H/E and H^3^/E^2^ ratios. The indentation depth was maintained at a level below 160 nm in order to minimize the influence of the substrate. The microstructural observations from the cross-sectional SEM images in Figure 1 and the bright-field TEM images in Figure 5 demonstrate that an increase in substrate bias voltage results in a progressive refinement of the columnar grain size. However, the grain size, internal stress, and defects collectively influence the surface hardness. The hardness exhibits an increase from 8.2 GPa at a bias voltage of −25 V to 16.5 GPa at −50 V, followed by a slight decline to 15.7 GPa as the bias voltage rises to −75 V. A comparable pattern is evident in the elastic modulus, which rises from 136.3 GPa at −25 V to 187.4 GPa at −50 V, before exhibiting a slight decline to 180.5 GPa at −75 V. The results of the TEM and XRD analyses indicate that an increase in bias voltage is associated with a reduction in columnar grain size and an elevation in internal tensile stress. These two factors contribute to the slightly higher hardness observed at −50 V compared to −75 V. While hardness is traditionally regarded as a key determinant of wear resistance, the H/E ratio, which represents the ratio of hardness (H) to elastic modulus (E), is increasingly recognized as a more reliable predictor of wear resistance and resistance to elastic strain to failure [58,59]. A higher H/E ratio is often indicative of superior wear resistance in coatings [58]. Moreover, the H^3^/E^2^ ratio provides valuable insight into the material’s resistance to plastic deformation [59,60]. As illustrated in Figure 6b, both the H/E and H^3^/E^2^ ratios exhibit trends similar to those observed for hardness and elastic modulus. An optimized substrate bias voltage not only refines the film’s grain structure but also prevents excessive internal stress, thereby enhancing durability [61]. These findings indicate that CrSiN films deposited at a substrate bias voltage of −50 V demonstrate optimal performance, with superior wear resistance and plastic deformation resistance. This is evidenced by the H/E and H^3^/E^2^ ratios, which reach values of 0.088 and 0.128, respectively.

#### 3.4.2. The Micro-Scratch Test of CrSiN Films

**Figure 7:**
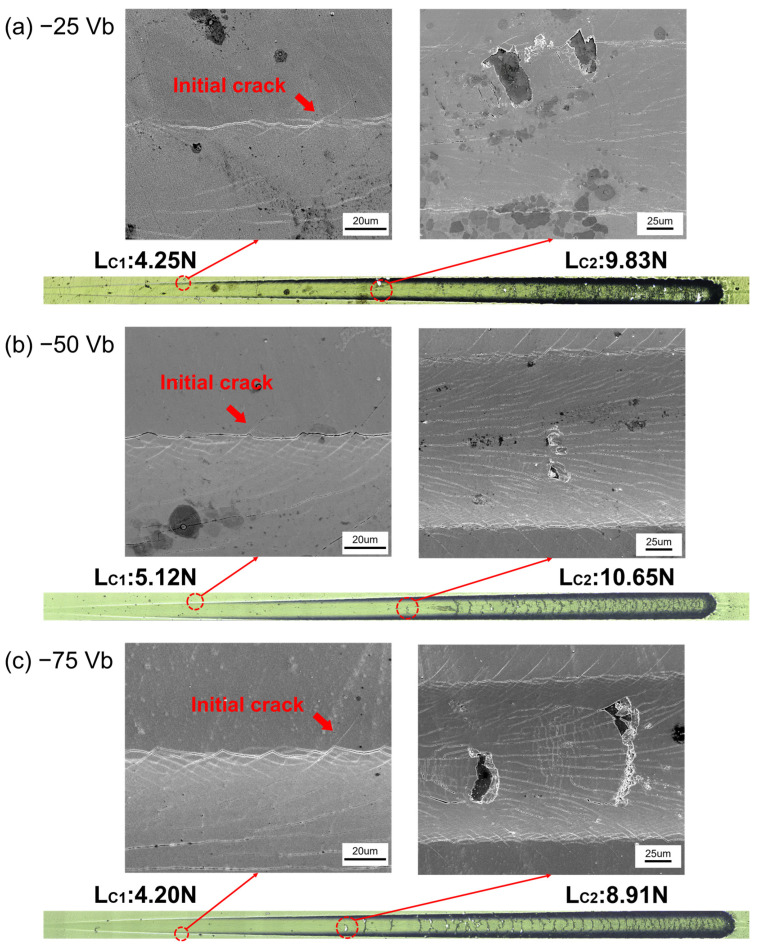
Scratch tracks and the resulting surface morphologies of (**a**) −25 Vb, (**b**) −50 Vb, (**c**) −75 Vb CrSiN films.

In order to evaluate the adhesion properties of the CrSiN films under varying substrate bias voltages, scratch tests were conducted. The optical micrographs of the scratch tracks, along with the corresponding critical loads, are presented in Figure 7. The critical loads, L_C1_ and L_C2_, were employed to assess the adhesion quality of the films to the substrate, and are also presented in Table 3. As defined in previous studies [62,63], the critical loads, L_C1_ and L_C2_, represent the onset of crack initiation and coating detachment, respectively. A higher critical load value indicates superior adhesion performance.

As illustrated in Figure 7a, the CrSiN films deposited at a substrate bias voltage of −25 V exhibited the initiation of microcracks on both sides of the scratch track when the applied load reached 4.25 N, which corresponds to the L_C1_ value for this sample. As the indenter advanced, the microcracks propagated. Upon increasing the load to 9.83 N, the coating began to detach from both sides of the groove, accompanied by deformation and lateral material accumulation. This defined the L_C2_ value. In comparison, when the substrate bias voltage was increased to −50 V, the films demonstrated improved adhesion properties, with L_C1_ and L_C2_ values increasing to 5.12 N and 10.65 N, respectively, as shown in Figure 7b. However, an increase in the substrate bias voltage to −75 V resulted in a reduction in the initial failure load (L_C1_) to 4.20 N, while coating deformation and lateral accumulation were observed at 8.91 N (L_C2_), indicating the poorest crack resistance among the tested conditions. Although the H/E and H^3^/E^2^ values of the −75 V CrSiN films were higher than those of the −25 V CrSiN films, the stress of the films under different substrate bias voltages influenced the adhesion strength of the film–interlayer–substrate assembly [61]. Nevertheless, the scratch test results demonstrate that optimizing the substrate bias voltage is an effective strategy to enhance adhesion properties.

#### 3.4.3. Friction and Wearing Properties

**Figure 8:**
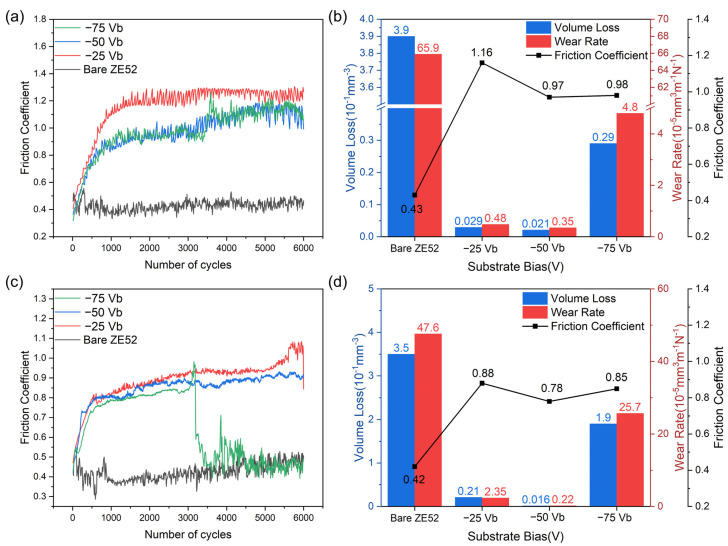
Shows the results of the pin-on-disk test: (**a**,**c**) friction coefficient as a function of the number of cycles, and (**b**,**d**) plots for volume loss, wear rate, and friction coefficient of CrSiN films deposited under different substrate bias voltages. The tests were conducted with a load force of (**a**,**b**) 2 N and (**c**,**d**) 4 N.

Figure 8 illustrates the friction coefficient versus the number of cycles for CrSiN films at varying substrate bias voltages under 2 N and 4 N load pin-on-disk wear tests. The quantitative data pertaining to the wear volume loss, wear rate, and wear width are presented in Table 4. Figure 8a depicts the outcomes of the 2 N load wear test, which demonstrates that the friction coefficients of all CrSiN films exhibit a gradual increase with the number of cycles. It is noteworthy that as the substrate bias voltage increases from −25 V to −50 V, the friction coefficient decreases. However, for the samples with a −75 V substrate bias, a slight increase in the friction coefficient is observed. This trend is consistent with the observations made using atomic force microscopy (AFM). The CrSiN films deposited at −25 V display a surface with a greater degree of roughness, while those deposited at −50 V exhibit a smoother surface. In contrast, the application of a substrate bias of −75 V results in a more intense ion bombardment, which in turn gives rise to the formation of surface defects and an increase in surface undulations. These factors contribute to a slight increase in the friction coefficient for the −75 V samples in comparison to the −50 V samples. With regard to the wear volume loss and wear rate illustrated in Figure 8b, a slight decrease is observed in both measures as the bias voltage increases from −25 V to −50 V. However, a notable rise is evident at −75 V, which can be attributed to the poorest adhesion behavior, as evidenced by the results of the scratch test. The CrSiN films deposited at −50 V exhibit the lowest wear volume loss (2.1 × 10^−3^ mm^3^) and wear rate (3.5 × 10^−6^ mm^3^·m^−1^·N^−1^), superior to that of other films. Furthermore, all CrSiN films, irrespective of substrate bias voltage, exhibit lower wear volume loss and wear rate than the bare ZE52 magnesium alloy. This evidence substantiates the assertion that CrSiN films demonstrate robust adhesion to the ZE52 magnesium alloy, thereby enhancing the tribological behavior under a 2 N load.

Figure 8c illustrates the results of the wear test conducted under a 4 N load. The CrSiN films deposited at −50 V exhibited a notable reduction in the friction coefficient, wear volume loss, and wear rate in comparison to the −25 V and −75 V CrSiN films. Although the −25 V CrSiN films display commendable wear resistance when subjected to a 2 N load, it is unable to withstand the tungsten carbide grinding balls when the load is increased to 4 N. After approximately 5500 cycles, the friction coefficient of the −25 V CrSiN films begin to exhibit pronounced fluctuations, with a notable decline observed around 5900 cycles. Similarly, the −75 V CrSiN films exhibit this pronounced fluctuation at an earlier point in their operational lifetime, at approximately 3000 cycles. This behavior can be attributed to the previously discussed cause: the −75 V CrSiN films exhibit the poorest adhesion behavior, rendering them more susceptible to cracking under a 4 N load compared to a 2 N load. It is postulated that the −25 V and −75 V CrSiN films, which serve as protective layers, have undergone severe wear and started to peel off, resulting in repeated grinding of the film’s interior. This is evidenced by the subsequent wear track profile. In contrast, the −50 V CrSiN films demonstrate a consistent increase in the wear coefficient with each wear cycle, maintaining a well-structured thin film throughout the wear tests. This results in the lowest friction coefficient, wear volume loss (1.6 × 10^−3^ mm^3^), and wear rate (2.2 × 10^−6^ mm^3^·m^−1^·N^−1^) compared to the other two films with varying bias voltages.

**Figure 9:**
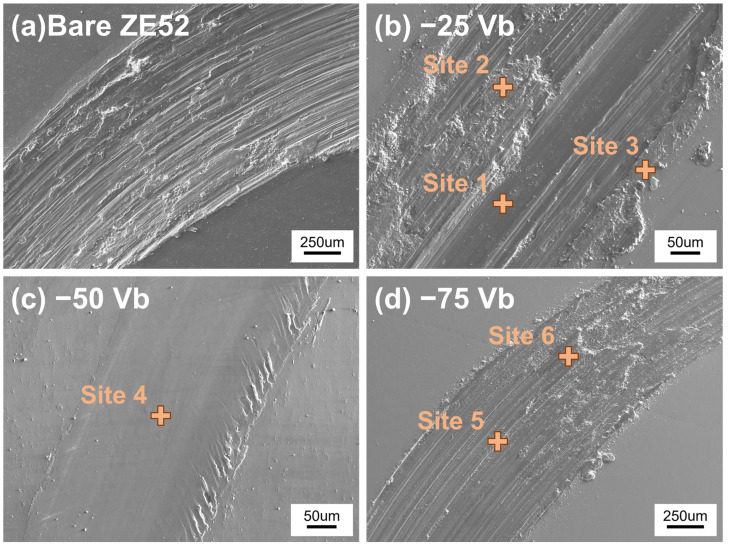
SEM images showing the wear track morphologies under loading force of 4 N. (**a**) bare ZE52 substrate, (**b**) −25 Vb, (**c**) −50 Vb, and (**d**) −75 Vb CrSiN films.

Figure 9 depicts SEM images of the wear tracks under a loading force of 4 N, and the corresponding quantitative analysis of the wear tracks is presented in Table 5. As illustrated in Figure 9a, the uncoated ZE52 substrate exhibits considerable surface deterioration, accompanied by a notable accumulation of debris following the wear test. The width of the wear track was found to be 1184 μm, which is larger than that observed in other CrSiN films. Figure 9b depicts the wear width of the CrSiN film deposited under a substrate bias voltage of −25 V, which was evaluated at 307 μm, with evidence of partial delamination of the CrSiN film. Despite the −25 V CrSiN film being the thickest of all the sputtered films, its loose structure and large columnar grains resulted in poor adhesion to the ZE52 substrate, as illustrated in Figure 7. The WDS analysis at site 1 in Figure 9b revealed the presence of Mg, Zn, Y, and Gd, confirming that portions of the protective film had worn away, exposing the substrate. In contrast, analysis of site 2 revealed the presence of Cr, Si, and N, indicating that a portion of the CrSiN film remained intact even after 6000 cycles. This finding is in accordance with the results presented in Figure 8c. The peeling process is believed to have commenced at approximately 5900 cycles. While the wear test concluded at 6000 cycles, the peeling was incomplete, leaving residual sputtered film on the substrate. An additional site, designated as 3, was identified at the periphery of the wear track. This site exhibited the presence of Cr, Si, N, Mg, W, and O. It is notable that the region situated beyond the wear fringes was not directly subjected to the wear process. However, the grinding action of the tungsten ball, CrSiN film, and ZE52 substrate resulted in the accumulation of debris along the wear track. It is possible that some of the magnesium debris from the substrate underwent oxidation as a result of the high-temperature friction, which would explain the higher contents of Mg, W, and O compared to areas not subjected to the wear test. As illustrated in Figure 9c, the width of the wear track for the −50 V CrSiN film decreased to 280 μm, exhibiting no signs of peeling. The WDS result at site 4 revealed the presence of Cr, Si, and N. A comparison of the two previous CrSiN films revealed that the −75 V CrSiN film exhibited the widest wear track width, reaching 901 μm (Figure 9d). The WDS results indicated that there was barely any Cr, Si, and N content remaining at sites 5 and 6. Conversely, the majority of the composition at these sites was Mg, which signified a significant depletion of the −75 V CrSiN layer. This phenomenon may be attributed to a combination of factors, including increased ion bombardment during deposition, which may have disrupted the microstructure, or the formation of stress concentration within the film, which may have reduced its overall durability under tribological conditions. The microstructural analysis of the −75 V CrSiN film indicates that, despite its comparable or higher hardness compared to films deposited under lower bias conditions, its adhesion and toughness appear to be significantly compromised. The wear track morphology suggests a brittle fracture of the coating, likely exacerbated by the internal stress profile induced during the deposition process. Additionally, the SEM images demonstrate a more fragmented wear debris pattern, indicative of coating delamination and catastrophic failure.

### 3.5. Corrosion Properties of CrSiN Coatings

**Figure 10:**
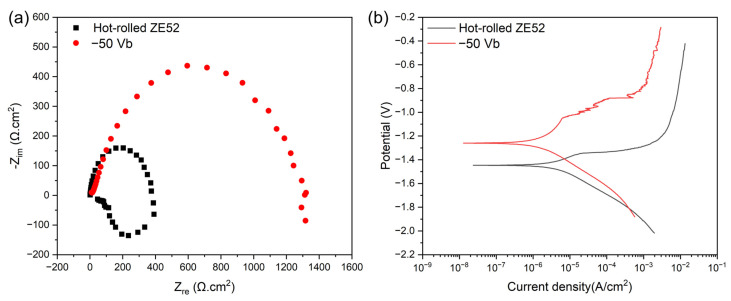
(**a**) EIS and (**b**) PDP curves for the bare ZE52 substrate and −50 Vb CrSiN films in a 3.5 wt% NaCl aqueous solution.

Figure 10 depicts the electrochemical impedance spectroscopy (EIS) and potentiodynamic polarization (PDP) measurements for the bare ZE52 and −50 V CrSiN films in a 3.5 wt% NaCl aqueous solution at room temperature. The polarization impedance (Rp), corrosion potential (E_corr_), and corrosion current density (I_corr_) were obtained through the Tafel extrapolation method, with the corresponding quantitative values provided in Table 6. As illustrated in Figure 10a, the EIS data, presented as a Nyquist plot, allow for the evaluation of the corrosion properties of the samples by comparing the diameters of the semicircles in the plot [64]. The larger the diameter, the greater the corrosion resistance of the samples. The results demonstrate that depositing the CrSiN films on the ZE52 substrate under a bias voltage of −50 V markedly enhances corrosion resistance, attaining values approximately three times those of the bare ZE52 substrate. Figure 10b illustrates that the CrSiN films deposited under a bias voltage of −50 V exhibit a lower corrosion current density in comparison to the bare ZE52 substrate, with values of 1.2255 × 10^−6^ (A/cm^2^) and 3.1324 × 10^−6^ (A/cm^2^), respectively. A reduction in corrosion current density is indicative of diminished charge transfer, which consequently enhances corrosion resistance [65]. This finding is in accordance with the results obtained from the EIS analysis. In conclusion, the deposition of the CrSiN films under a substrate bias voltage of −50 V on the ZE52 substrate effectively improves the overall corrosion resistance.

## 4. Conclusions

This study successfully demonstrated the enhancement of wear and corrosion resistance of ZE52 magnesium alloy by depositing CrSiN films via DC magnetron sputtering with a CrSi buffer layer. The critical results are as follows:

1. An increase in the substrate bias voltage from −25 V to −50 V resulted in a refinement of the CrSiN film’s columnar grain structure, leading to a denser and smoother surface. However, an excessive bias voltage of −75 V introduced defects and increased roughness.
2. The CrSiN films deposited at −50 V exhibited the highest hardness (16.5 GPa) and elastic modulus (187.4 GPa), providing the best mechanical strength and resistance to plastic deformation. In comparison, the CrN PVD coatings on AZ31hp magnesium alloys, as reported by Hollstein et al. [19], exhibited a hardness of approximately 12 GPa. This indicates that, when applied to magnesium alloys, CrSiN coatings offer superior mechanical properties compared to CrN coatings, showcasing their potential for enhanced performance.
3. The −50 V CrSiN films demonstrated the lowest wear rate and friction coefficient under both 2 N and 4 N loads, outperforming other conditions and the bare ZE52 substrate. Conversely, the −75 V films exhibited inferior wear resistance due to poor adhesion.
4. The electrochemical tests demonstrated that the −50 V CrSiN films markedly enhanced the corrosion resistance of the ZE52 alloy, resulting in a markedly lower corrosion current density (1.2255 × 10^−6^ A/cm^2^) and a markedly higher polarization resistance (Rp = 1271.4 Ω·cm^2^), which was approximately three times greater than that of the bare substrate.

In conclusion, optimizing the deposition parameters of CrSiN coatings, particularly the substrate bias voltage, is crucial for enhancing wear and corrosion resistance. Among the tested conditions, the CrSiN films deposited at −50 V demonstrated the best overall performance, exhibiting improved mechanical, tribological, and electrochemical properties. These findings suggest that the −50 V CrSiN films are a promising solution for industrial applications requiring durable magnesium alloys. Furthermore, CrSiN coatings are not only limited to magnesium alloys; they can also be applied to materials such as tool steel or stainless steel, serving as a hard protective layer to enhance surface durability [66]. This versatility highlights the potential for expanding the industrial applications of CrSiN coatings, offering innovative solutions for sectors demanding high-performance protective materials. To further enhance the properties of CrSiN coatings, a multilayer structure could be employed. As demonstrated in the study conducted by Lin et al. [67], the performance of multilayer coatings, such as (CrN/Cr)x, improves with an increasing number of layers. Specifically, the hardness, wear resistance, and polarization resistance showed significant enhancement as the number of CrN/Cr layers increased. This approach provides a promising avenue for developing advanced coatings with superior mechanical and electrochemical properties, thereby extending their applicability across various demanding industrial environments.

## Figures and Tables

**Table 1:** Thickness and surface roughness of CrSiN films deposited at different substrate bias voltages.

Sample Number	Temperature (°C)	Bias Voltage (V)	Thickness (μm)		Ra Roughness (nm)
			CrSiN/CrSi	Total	
−25 Vb	250	−25	2.84/0.33	3.17	11.6
−50 Vb	250	−50	2.62/0.32	2.94	3.95
−75 Vb	250	−75	2.38/0.34	2.71	4.58

**Table 2:** The chemical compositions of CrSiN films deposited under different substrate bias voltages.

Sample Number	Temperature (°C)	Bias Voltage (V)	Compositions (at%)			
			Cr	Si	N	O
−25 Vb	250	−25	46.92	5.80	46.6	0.68
−50 Vb	250	−50	46.85	5.76	45.86	1.53
−75 Vb	250	−75	45.94	5.69	45.56	2.81

**Table 3:** Hardness, Elastic Modulus, H/E ratio, and H^3^/E^2^ ratio of different condition CrSiN films.

Sample Number	Hardness (GPa)	Elastic Modulus (GPa)	H/E	H^3^/E^2^ (GPa)	L_C1_ (N)	L_C2_ (N)
−25 Vb	8.2 ± 0.14	136.3 ± 2.89	0.060	0.030	4.25	9.83
−50 Vb	16.5 ± 0.13	187.4 ± 2.71	0.088	0.128	5.12	10.65
−75 Vb	15.7 ± 0.37	180.5 ± 2.90	0.087	0.119	4.20	8.91

**Table 4:** Volume loss and wear rate of CrSiN films deposited at varying substrate bias voltages under a loading force of 2 N and 4 N.

	2 N			4 N		
	Volume Loss (10^−1^ mm^3^)	Wear Rate (10^−5^ mm^3^ m^−1^ N^−1^)	Wear Width (μm)	Volume Loss (10^−1^ mm^3^)	Wear Rate (10^−5^ mm^3^ m^−1^ N^−1^)	Wear Width (μm)
Bare ZE52	3.9	65.9	1099	3.5	47.6	1184
−25 Vb	0.029	0.48	251	0.21	2.35	307
−50 Vb	0.021	0.35	244	0.016	0.22	280
−75 Vb	0.29	4.8	256	1.9	25.7	901

**Table 5:** WDS quantitative analysis of selected sites in the wear tracks of CrSiN films with different substrate bias voltages.

Sample Number	Site	Compositions (at%)										
		O	C	W	Cr	Si	N	Mg	Zn	Y	Nd	Gd
−25 Vb	1	0.30	0.51	0.00	0.38	0.06	0.89	96.37	1.19	0.24	0.00	0.05
	2	0.42	0.58	0.22	44.88	6.01	47.28	0.36	0.24	0.00	0.00	0.00
	3	2.61	0.54	1.17	43.10	5.85	43.42	3.09	0.22	0.00	0.00	0.00
−50 Vb	4	0.96	0.45	0.02	46.67	5.38	46.35	0.00	0.14	0.02	0.00	0.00
−75 Vb	5	1.26	0.58	0.01	0.03	0.02	0.00	96.58	1.36	0.12	0.00	0.00
	6	9.07	2.80	0.02	0.37	0.06	0.09	85.26	1.93	0.30	0.03	0.08

**Table 6:** Rp, I_corr_, and E_corr_ for the bare ZE52 substrate and −50 Vb CrSiN films in a 3.5 wt% NaCl aqueous solution.

	Rp (Ω·cm^2^)	I_corr_ (A/cm^2^)	E_corr_ (V)
Bare ZE52	381.36	3.1324 × 10^−6^	−1.447
−50 Vb	1271.4	1.2255 × 10^−6^	−1.260

## Data Availability

The original contributions presented in the study are included in the article, further inquiries can be directed to the corresponding author.
